# How can we achieve health equity? Revisit the premise informing the scientific method

**DOI:** 10.1111/hex.13369

**Published:** 2022-02-16

**Authors:** Karen L. Fortuna, Matthew F. Hudson, Amanda Myers, Arya Kadakia, Jennifer Rivera, Tony Nutz

**Affiliations:** ^1^ Department of Psychiatry Geisel School of Medicine at Dartmouth Hanover New Hampshire USA; ^2^ Prisma Health Greenville South Carolina USA; ^3^ Brandeis University Waltham Massachusetts USA; ^4^ Dartmouth College Hanover New Hampshire USA; ^5^ Boston College School of Social Work Boston Massachusetts USA; ^6^ National Alliance on Mental Illness Wichita Kansas USA

The advent of the COVID‐19 vaccine may lead to longer and healthier lives via reduced virus transmission and the gradual abatement of the pandemic. Yet, vaccines and evidence‐based information about them are differentially accessible among some populations, notably racial and ethnic disadvantaged groups, rural community residents, people with disadvantaged socioeconomic resources and individuals with disabilities. For example, the Center for Disease Control and Prevention estimates that as of March 2021,^1^ Native Americans suffer from more COVID‐19‐attributed mortality than any other group and are 2.4 times more likely to die than White, non‐Hispanic persons. Similarly, Hispanic or Latino persons and African Americans are, respectively, 2.3 and 1.9 times more likely to die from COVID‐19. Apart from racial and ethnic disparities, individuals with a serious mental illness are likely to die of COVID‐19. For instance, individuals diagnosed with schizophrenia are nearly three times more likely to suffer COVID‐19‐attributed mortality than those without the disorder. A first step to address such health inequities requires explicitly examining the existing power structure of the healthcare system. This reflection requires carefully critiquing the premise informing its scientific method.

Historically, medicine ascribes to reductionism, which posits that individuals can address complexity by separating challenges into fundamental elements. Reductionism motivates the current ethos of quality improvement and application in science and even permeates the continuum of medical research and practice—including the diagnosis, treatment and prevention of diseases. It nurtures the idea of an *expert* self with a highly specialized focus on risk aversion who learns to implement short‐term solutions that rapidly return the body to homeostasis. Reductionism profoundly influences science and has informed many medical advancements, including the COVID‐19 vaccines. However, reductionism in healthcare lacks the necessary characteristics to *engage* disadvantaged groups—humility, sustained commitment and a willingness to relinquish expert status by acknowledging the lived experience of marginalized stakeholders.

In comparison, a humanistic philosophical approach to science (commonly found within the social sciences) can possibly elucidate health inequalities by promoting community partnerships. Humanism embraces the belief in the freedom of the human mind and the basic equality of all human minds. Thus, such research prioritizes the human experience and promotes the inclusion of disadvantaged populations as partners in research. Today's climate of political and social unrest, coupled with a pandemic exposing care and wellness inequality, highlights the limitations of using traditional, hierarchical team approaches that intervene on behalf of—but *not with*—disadvantaged populations. Circumstances now require methodological pluralism that integrates aspects of humanism to advance partnerships in medical research and produce superior clinical outcomes that are better aligned with the priorities, assumptions, strengths and limitations of the disadvantaged population.

During public health emergencies like COVID‐19, community partnerships with disadvantaged groups encourage feasible outbreak control solutions that enhance quality of life. For example, Coady et al.^2^ partnered with community members in New York City's East Harlem and the Bronx to develop an intervention that promoted influenza vaccination uptake for socially isolated groups (e.g., people who use illicit substances, immigrants, older adults, sex workers and people experiencing homelessness) who can suffer fatal effects from the flu. Community partnership activities included information dissemination (e.g., leaflets, comic strips and presentations) with local organizations for low‐income communities. Similarly, Lee et al.^3^ developed an HPV vaccine text messaging‐based intervention for Korean Americans using community‐based participatory research. Informed by a representative community advisory board, focus groups and usability studies, the partnership developed valuable content and recruitment strategies for intervention.

These activities are merely two successful examples of a humanism‐informed approach towards science that increased vaccination compared to previous years. Accordingly, addressing the needs of historically disadvantaged populations in medicine requires balancing both reductionist and humanistic approaches. We encourage three humanistic‐imbued practices in medicine that can help dismantle health inequities and improve the well‐being of disadvantaged populations.

## 1 FUNDAMENTALLY EXPLORE AND CHALLENGE THE EPISTEMOLOGY MOTIVATING PROBLEM IDENTIFICATION

Many traditional postgraduate programmes fail to intentionally consider the humanistic approach, despite its presumed advantage. We suggest that the curricula should also explore the role of epistemology in health inequities and should extend beyond implementing a set of scientific research methodologies (e.g., oversampling a specific population or mandating a minimum number of female participants). Humanistic approaches require the gradual, continuous process of deconstructing and reconstructing the self to understand and confront our transgressions, embrace allyship and challenge our implicit biases. This introspection is essential for clinical care and research.

## 2 PROMOTE ACCOUNTABILITY THROUGH THE CONTINUOUS APPRAISAL OF CLINICAL AND COMMUNITY PARTNERSHIPS

Just as learning health systems encourage continuous quality improvement, so should these same systems encourage humanistic approaches to address health disparities. Methods to examine the strength of clinical and community partnerships commonly rely on *retrospective* accounts of stakeholders' experience working with researchers—including qualitative interviews, focus groups and process outcomes such as research training, grants funded and publications. Such methods may only inform participatory medicine practices post hoc, thus depriving partnerships of feedback for real‐time improvement. Conducting surveys designed to facilitate readability and understandability for individuals with potential low health literacy at multiple time points throughout a study may facilitate patient‐reported perspectives and offer opportunities for continuous improvement of the partnership and ongoing accountability.

## 3 SELECT THE APPROPRIATE PARTICIPATORY APPROACH TO MEDICINE FOR THE POPULATION OF INTEREST

It is essential to select the appropriate participatory approach for the population of interest. While multiple models of participatory medicine exist (e.g., community‐based participatory research, Active Community Engagement Continuum, Rapid Assessment, and Response Evaluation, Diffusion of Innovations), researchers commonly use approaches that have been successful among nondisadvantaged populations for disadvantaged groups, naturally leading to problems in accomplishing the desired results. Equity‐based approaches require greater stakeholder involvement with the decision‐making and research activities at all stages of research—including observation, problem definition, hypothesis development, testing and revision—to produce relevant results and wide‐scale uptake. Thus, collaborating with disadvantaged populations utilizing the appropriate participatory framework can elevate our capacity to address health disparities.

These three practices are necessary first steps to address health inequities. It is essential for all stakeholders, including clinicians, policy‐makers, academia and researchers, to confront their scientific biases and implement a framework that promotes community partnerships in every stage of their work. These steps require a balance of both reductionist and humanistic approaches to encourage a more informed approach towards problem identification and problem solving in its scientific method. Such applied curiosity may encourage scientists and patients alike to collectively develop novel insights informing intervention innovation in service of health equity.

## FUNDING INFORMATION

Karen L. Fortuna was funded by an NIMH K01 award (K01MH117496).

## AUTHOR CONTRIBUTIONS

All authors contributed equally to the production of this editorial. While Karen L. Fortuna, Matthew F. Hudson and Amanda Myers conducted research and wrote the initial content, Arya Kadakia, Jennifer Rivera and Tony Nutz provided lay perspectives and also contributed to the editing of the article by simplifying, restructuring and rewriting various components.

## Data Availability

No additional data were used in the production of this editorial.
